# The role of prognostic nutritional index in predicting amputation in patients with lower extremity peripheral artery disease

**DOI:** 10.34172/jcvtr.2021.02

**Published:** 2021-01-13

**Authors:** Hilal Erken Pamukcu, Hamza Sunman, Alperen Taş, Mert Aker, Haluk Furkan Şahan, Sadık Açıkel

**Affiliations:** ^1^Ministry of Health, University of Health Sciences, Dışkapı Yıldırım Beyazıt Training & Research Hospital, Department of Cardiology, Ankara, Turkey

**Keywords:** Prognostic Nutritional Index, Lower-Eextremity Peripheral, Artery Disease, Amputation

## Abstract

***Introduction:*** Lower-extremity peripheral artery disease (PAD) can lead to a wide spectrum of symptoms that can progress from claudication to amputation. The prognostic nutritional index (PNI), which is calculated using the levels of albumin and lymphocyte, is an accepted indicator of immunological and nutritional status. In this study, the association between nutritional status determined using the PNI, and extremity amputation in patients with lower-extremity PAD was investigated.

***Methods:*** Lower-extremity PAD patients who had been admitted to the cardiology clinic of the Dışkapı Yıldırım Beyazıt Training & Research Hospital with stage 2b or higher claudication, and who were technically unsuitable for revascularization or underwent unsuccessful revascularization procedure were enrolled in this retrospective study. Patients were grouped according to whether or not limb amputation had been performed previously. Potential factors were tested to detect independent predictors for amputation with logistic regression analysis.

***Results:*** A study group was formed with 266 peripheral artery patients. The amputated group (39 patients) had a higher number of hypertensive (76.9% vs 57.7%; *P* = 0.032) and diabetic (92.3% vs 54.2%; *P* <0.001) patients than those in the non-amputated group (227 patients). The median PNI value of the amputated group was lower than that of the non-amputated group (31.8 vs 39.4; *P* <0.001). Multivariate logistic regression showed that the PNI (OR: 0.905, 95% CI: 0.859 – 0.954; *P* <0.001) was independently related with amputation.

***Conclusion:*** Immune-nutritional status based on PNI was independently associated with limb amputation in patients with lower-extremity PAD.

## Introduction

Peripheral artery disease (PAD) is a condition characterized by narrowing of the artery diameter, which affects the entire arterial system, except the cerebral and coronary systems.^1–3^ Lower-extremity atherosclerotic PAD is the most common clinical presentation in daily practice. The classic risk factors of atherosclerotic disease are often present in these patients. Many studies have shown that all-cause mortality and cardiovascular mortality and morbidity (myocardial infarction (MI), stroke) were increased in those with lower-extremity PAD, even after adjustment for the classical atherosclerosis risk factors.^4^

The most serious manifestation is critical limb ischemia, which has been observed in 11% of peripheral artery patients^5^, and the 1-year mortality rate can reach 10%–40%, as can the risk of limb loss (10%–40%)^6^. In patients with critical limb ischemia, amputation rates have fallen recently with revascularization methods and medical treatment, but especially in patients with no chance of revascularization, increased awareness and improvements in wound care^7^ are of paramount importance.^6^

The importance of nutrition is known in chronic wound healing.^8,9^ Nutrition disorders delay wound healing by prolonging the inflammatory process, and reducing fibroblast proliferation and collagen synthesis.^8,10^ The prognostic nutritional index (PNI) is a scoring system that reflects the immunological and nutritional status, and is calculated using the levels of albumin and lymphocytes.^11^ Recent studies have shown the PNI as a predictor of many types of cancer,^12,13^ MI,^11^ and heart failure mortality.^14^ In this study, it was aimed to evaluate the association between nutritional status, as determined by PNI, and extremity amputation in patients with lower-extremity PAD.

## Materials and Methods

Between November 2017 and November 2019, lower-extremity PAD patients who had applied to the cardiology clinic of the Dışkapı Yıldırım Beyazıt Training & Research Hospital were included in this retrospective study.

Eligibility criteria for enrollment in the study included having lower-extremity PAD with stage 2b or higher claudication according to the Fontaine classification,^15^ being technically unsuitable for revascularization, or having experienced an unsuccessful revascularization procedure previously.

Patients with a body mass index (BMI) below 18 kg/m^2^, acute or chronic infection, liver failure, kidney disease, chronic inflammatory disease, and heart failure were excluded from the study. A blood pressure value of ≥140/90 mm Hg or the use of antihypertensive drugs was used for the diagnosis of arterial hypertension.^16^ Criteria for the diagnosis of diabetes mellitus (DM) included having a fasting blood glucose level of 126 mg/dL or 200 mg/dL of glucose in the blood sample taken at any time, or using antidiabetic drugs.^17^

PAD was defined as an ankle-brachial index (ABI) ≤0.90 and then confirmed by conventional or computed tomography angiography with stenosis of more than 50% of the vessel diameter. Normal ABI was defined as 1.00 to 1.30^1^. The patients were grouped as those who underwent amputation and those who did not. The patient data were tested to determine the independent predictors of amputation.

Patients were also grouped according to lesion localization as above the knee, below the knee, and diffuse involvement. External iliac, common, and superficial femoral and popliteal artery lesions were considered as above-knee (supragenicular), while posterior tibial, anterior tibial, dorsalis pedis artery lesions were considered as below-knee (infragenicular) involvement. Those with both above-knee and below-knee involvement were accepted as diffuse involvement.

The results of the biochemical tests and complete blood counts from peripheral venous blood samples taken from all of the patients were used in the study. PNI values, which were used as a nutritional marker, were calculated using the formula: 10 × serum albumin (g/dL) + 0.005 × total lymphocyte count (per mm^3^), in all of the patients.^18^

Transthoracic echocardiographic examination was performed using a Philips Epic 5 (Philips Healthcare, Andover, MA, USA) instrument with a 1-5 MHz transducer. Parasternal long- and short-axis views, and apical 2- and 4-chamber views were obtained. The left ventricular (LV) and left atrial (LA) diameters were measured from the M-mode images in parasternal long axis view. The modified Simpson method was used to calculate the left ventricle ejection fraction using the apical 4-chamber views.^19^

### Statistical analysis

Statistical evaluation was conducted with the software Statistical Package for Social Sciences (SPSS) for Windows 20 (IBM SPSS Inc., Chicago, IL, USA). The normality of the distribution of data was analyzed with Kolmogorov-Smirov test . Categorical data were presented as number and percentage. Continuous variables were presented as the mean ± standard deviation (SD) when normally distributed, and as the median and interquartile ranges otherwise. Differences between the groups were evaluated using the Student *t* test for normally distributed variables and Mann-Whitney *U* test for variables without normal distribution. The chi-square test was used to compare the categorical variables as appropriate. Univariate logistic regression analysis was used to evaluate the relationship between the variables and the presence of limb amputation. Variables that were statistically significant in the univariate analysis were further used in a multivariate logistic regression analysis with the enter method, in order to determine the independent predictors of limb amputation. The results of the regression analyses were presented as odds ratios (ORs), 95% confidence intervals (CIs) and *P* values. The receiver operating characteristic (ROC) curve analysis was used to establish the optimum cut-off level of the PNI to predict limb amputation. A *P* < 0.05 was also considered as statistically significant. Post-hoc power analysis, comparing the PNI values between the 2 groups, showed a power of more than 90% at a confidence interval of 95%.

## Results

In this retrospective study, a total of 868 patients with lower-extremity PAD who had applied to the cardiology clinic of the Dışkapı Yıldırım Beyazıt Training & Research Hospital were evaluated. The study group consisted of 266 peripheral artery patients after the exclusion criteria were evaluated.

All of patients were grouped according to whether they had been amputated previously. The amputated group comprised 39 (15%) patients, while the non-amputated group comprised 227 (85%) (Table 1). There were no statistically significant differences between the two groups in terms of age, gender, and smoking status. The proportions of patients with hypertensive (76.9% vs 57.7%, *P* = 0.032), and diabetic (92.3% vs 54.2%, *P* < 0.001) were higher in the amputated than the non-amputated group. The median PNI value was lower in the amputated group than the non-amputated group (31.8 (26–40) vs. 39.4 (36–42), *P* < 0.001).

**Table 1:** The Baseline study parameters of patients according to limb amputation

**Parameters**	**Amputated** **n = 39 (15%)**	**Not amputated** **n = 227(85%)**	***P*** ** value**
Age, years	67.3±11	65.5±9.9	0.300^a^
Female gender, n (%)	5(12.8%)	44(19.4%)	0.381^b^
Hypertension, n (%)	30(76.9%)	131(57.7%)	0.032^b*^
Diabetes mellitus, n (%)	36(92.3%)	123(54.2%)	<0.001^b*^
Smoking, n (%)	13(33.3%)	57(25.1%)	0.325^b^
Body mass index, kg/m^2^	23(23-24)	24(23-25)	0.209^b^
**Echocardiography parameters**			
LVEDD, mm	46(43-52)	48(43-51)	0.641^c^
LVESD, mm	24(19-28)	25(21-27)	0.601^c^
LA diameter, mm	39(35-42)	38(34-41)	0.379^c^
LV EF (%)	58(45-60)	60(45-60)	0.299^c^
Ascending aorta, mm	34(31-35)	33(32-36)	0.457^c^
**Laboratory parameters**			
Fasting glucose, mg/dL	158(122-200)	119(93-184)	0.009^c*^
BUN, mg/dL	54(34-95)	41(31-53)	0.001^c*^
Creatinine, mg /dL	1.13(0.87-1.67)	1(0.9-1.22)	0.306^c^
Sodium, mEq/L	136(133-138)	138(136-140)	<0.001^c*^
AST, U/L	20(16-33)	18(14-24)	0.141^c^
ALT, U/L	16(12-22)	16(10-23)	0.770^c^
Albumin, g/dL	3.1(2.5-3.9)	3.8(3.5-4.1)	<0.001^c*^
Hemoglobin, g/dL	11.3(9.3-13.5)	13.1(10.8-14.5)	0.002^c*^
WBC, cells/mL	9.1(7.3-11.8)	8.7(7.5-10.3)	0.351^c^
Platelet count, 109/L	265(222-336)	259(200-306)	0.262^c^
Lymphocyte count, 109/L	1.5(1.1-1.9)	1.9(1.4-2.4)	0.003^c*^
RDW	16.6(14.3-17.7)	14.6(13.7-16.1)	0.001^c*^
Total cholesterol, mg/dL	147(109-191)	165(126-204)	0.118^c^
Triglyceride, mg/dL	117(81-204)	145(100-195)	0.226^c^
HDL cholesterol, mg /dL	34(28-39)	37(32-45)	0.026^c*^
LDL cholesterol, mg /dL	113(72-133)	120(94-149)	0.059^c^
PNI	31.8(26-40)	39.4(36-42)	<0.001^*^

Abbreviations: LVEDD, left ventricular end-diastolic diameters; LVESD, Left ventricular end-systolic dimension; LA, left atrium; LVEF, left ventricular ejection fraction; BUN, blood urea nitrogen; AST, aspartate aminotransferase; ALT, alanine aminotransferase; WBC, White blood cell; RDW, red blood cell distribution width; HDL, High-density lipoprotein; LDL, Low-density lipoprotein: PNI, prognostic nutritional index ^a^Student *t* test; mean ± standart deviation ^b^Pearson chi-square; number and percent ^c^Mann-Whitney *U* test; median (interquartile ranges) *Statistically significant

The baseline characteristics and laboratory parameters of the patients according to lesion involvement are shown in Table 2. The PNI value was the lowest in the group with diffuse involvement. The amputation rate was highest (21.39%) in the diffuse involvement group and it was 14.5% in the infragenicular group.

**Table 2:** The Baseline Characteristics, laboratory parameters and amputation status of patients with peripheral artery disease according to PAD location

**Parameters**	**Supragenicular** **n = 61 (22.9%)**	**Infragenicular** **n = 69 (25.9%)**	**Diffuse** **n = 136 (51.1%)**	***P*** ** value**
Age, years	64.8±8.6	66±11.2	66±10.3	0.728^a^
Female gender, n (%)	11(18)	9(13)	29(21.3)	0.351^b^
Hypertension, n (%)	38(62.3)	49(71)	74(54.4)	0.068^b^
Diabetes mellitus, n (%)	27(44.3)	44(63.8)	88(64.7)	0.019^b*^
Smoking, n (%)	12(19.7)	20(29)	38(27.9)	0.401^b^
Body mass index (kg/m^2^)	24(23-25)	23(23-25)	23(23-25)	0.398 ^c^
**Laboratory parameters**					
Fasting glucose, mg/dl	113(93-148)	142(101-185)	129(97-196)	0.304 ^c^
BUN, mg/dl	41(34-48)	43(35-60)	42(31-56)	0.197 ^c^
Creatinine, mg /dl	1.01(0.9-1.3)	1.0(0.9-1.27)	1.01(0.9-1.3)	0.966 ^c^
Sodium, mEq/L	138(137-140)	138(135-140)	137(135-139)	0.072 ^c^
AST, U/L	18(15.7-27.5)	19(16-30)	18(16-26)	0.665 ^c^
ALT, U/L	15.5(11-26)	15(10-23)	16(10-20)	0.728 ^c^
Albumin, g/dL	4.0(3.7-4.3)	3.87(3.5-4.2)	3.62(2.9-4.1)	0.001^c*^
Hemoglobin, g/dL	14.1(12.4-14.6)	12.6(10.9-14.3)	12.2(10.3-14.5)	0.015^c*^
WBC, cells/mL	8.4(7.5-10)	8.1(7.4-10.6)	9.0(7.4-10.6)	0.304 ^c^
Platelet count, 109/L	237(170-290)	265(216-306)	265(207-331)	0.025 ^c*^
Lymphocyte count, 109/L	1.9(1.4-2.45)	1.8(1.4-2.3)	1.8(1.3-2.47)	0.682 ^c^
RDW	14.4(13.6-15.8)	15.2(13.7-16.7)	14.9(13.8-16.8)	0.095 ^c^
Total cholesterol, mg/dL	167(144.7-203)	165(140-212)	175(140-207)	0.977 ^c^
Triglyceride, mg/dL	145(103-192)	127(98-210)	146(91-209)	0.804 ^c^
HDL cholesterol, mg /dL	38.5(32-46)	37(31-43)	35.5(31-44.7)	0.251 ^c^
LDL cholesterol, mg /dL	111(93.7-142)	117(88-151)	122(91-143)	0.953 ^c^
PNI	41.4(37.5-43.6)	39.1(35.6-42.6)	37.8(31.6-42)	0.001^c*^
Amputation status, n (%)	0(0)	10(14.5)	29(21.39)	<0.001^b*^

Abbreviations: BUN, blood urea nitrogen; AST, aspartate aminotransferase; ALT, alanine aminotransferase; WBC, White blood cell; RDW, red blood cell distribution width; HDL, High-density lipoprotein; LDL, Low-density lipoprotein; PNI, prognostic nutritional index ^a^One-way Analysis of Variance; mean ±standart deviation ^b^Pearson chi-square; number and percent c: Kruskal Wallis test; median (interquartile ranges) *Statistically significant

Potential parameters that may be associated with amputation were first evaluated by univariate analysis. Hypertension, DM, blood urea nitrogen (BUN), sodium, hemoglobin, red cell distribution width, lesion location, and PNI were all found to be associated with amputation (Table 2). When a multivariate logistic regression analysis was constructed that included all of these significant univariate predictors, it was found that DM, lesion location, and PNI were independently related with amputation. Results of regression analysis are shown in Table 3.

**Table 3:** Univariate and Multivariate logistic regression analysis showing independent predictors of amputation

	**Univariate analysis**			**Multivariate regression analysis**		
	**OR**	**95% CI**	***P*** ** value**	**OR**	**95% CI**	***P*** ** value**
Hypertension	2.443	1.109-5.383	**0.027**	1.952	0.763-4.992	0.163
Diabetes mellitus	10.100	3.03-33.9	**<0.001**	4.840	1.240-18.888	0.023*
Glucose	1.002	0.998-1.006	0.403	-	-	-
BUN	1.008	1.003-1.014	**0.003**	1.004	0.998-1.009	0.233
Sodium	0.840	0.763-0.925	**<0.001**	0.904	0.821-0.995	0.040
Hemoglobin	0.779	0.670-0.905	**0.001**	0.990	0.887-1.104	0.852
RDW	1.155	1.023-1.304	**0.020**	1.018	0.872-1.190	0.819
HDL cholesterol	0.974	0.938-1.010	0.159	-	-	**-**
Diffuse involvement	3.006	1.631-5.540	**<0.001**	2.865	1.347-6.093	0.006*
PNI	0.895	0.853-0.938	**<0.001**	0.916	0.867-0.967	0.001*

Abbreviations: CI, confidence interval; OR, Odds ratio; BUN, blood urea nitrogen; RDW, red blood cell distribution width; PNI, prognostic nutritional index *Statistically significant

**Figure 1:**
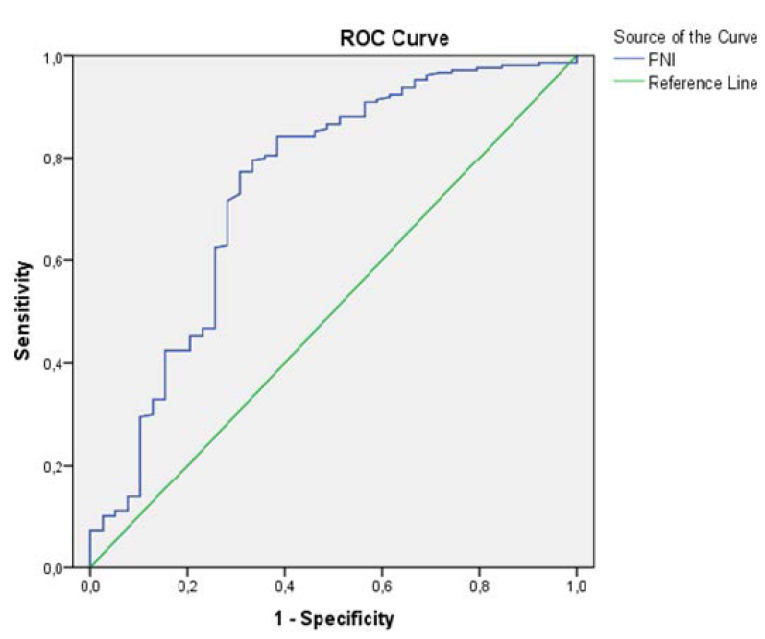
The receiver operating characteristic curves of prognostic nutritional index (PNI). PNI had an area under the curve (AUC) value of 0.741 (95% CI: 0.645-0.837; *P* <0.001)

The ROC curve analysis explored the discriminatory capability of the PNI for limb amputation. A PNI level 36.8 predicted limb amputation with a sensitivity of 71% and a specificity of 72% (AUC:0.741; 95% CI: 0.645-0.837; *P*<0.001) (Figure 1).

## Discussion

The present study demonstrated that immune-nutritional status was independently associated with limb amputation based on the PNI in patients with lower-extremity PAD.

The most common presentation of PAD is claudication. Complaints of patients with intermittent claudication may restrict their physical activities in their daily lives and progress to amputation in 5% of patients.^20,21^ Limb amputation, which develops as a result of critical limb ischemia, is the most serious condition that can be seen in the course of PAD, where the risk of 1-year limb loss can reach 20%, as can the 1-year mortality rate (20%).^22,23^

Limb amputation is a condition that may be necessary as the last option in the cases where there is no revascularization option or the patient does not respond to revascularization, leading to severe tissue loss, resulting in loss of function, morbidity, and mortality.

In addition to improvements in medical and interventional treatments, wound care improvements are of especially great importance in stopping the process leading to amputation. Additionally, nutritional status is very important, as malnutrition delays woundhealing. Malnutrition reduces collagen formation by reducing fibroblast proliferation, but also reduces angiogenesis.^24^ In the presence of malnutrition, T cell function, phagocytosis, and antibody levels decrease with increased risk of infection.^10^ As a result, tissue healing is delayed and the risk of limb loss is increased.

In the case of malnutrition, atrophy develops in lymphatic tissues, cellular immunity is disrupted, and bacteriolytic leukocyte activity is reduced. Damage to lymphocytes in the thymus and thymus atrophy occur. Interleukin (IL) metabolism, especially IL-1 activity, has been suppressed in malnutrition. Impaired IL-1 activity leads to a decrease in the production rate of lymphocytes.^25^ The PNI is a marker calculated simply by using the blood lymphocyte level and serum albumin level, and has been used in various studies as an indicator of nutritional and immunological status^11^. The PNI has been highlighted as a prognostic factor in many malignancies and chronic diseases.^26-28^

When the relationship between amputation and albumin, and lymphocyte count and the PNI itself was examined, the PNI was found to be more significantly associated with amputation. It is uncertain whether the nutritional status determined by the PNI in patients with need for amputation was a factor or a marker, because the clinical condition of these patients was worse. In peripheral artery patients requiring amputation, the PNI value may be low due to chronic wound inflammation, or the presence of malnutrition. In a previous study with patients receiving peritoneal dialysis, low albumin levels were attributed to malnutrition and inflammation.^29^ In a study showing that low PNI in heart failure was associated with increased mortality, malabsorption and chronic inflammation were considered responsible for the mechanism. In that study, malabsorption was thought to be related to cardio-intestinal interaction and this was the cause of cardiac cachexia.^30^ In peripheral artery patients, accompanying mesenteric artery ischemia may play a role in malnutrition by disrupting intestinal absorption. As general information, while the lymphocyte count, which is one of the components of the PNI, is expected to increase substantially in chronic inflammation, it is believed that the decrease in these patients suggested that the role of malnutrition onto the metabolism of lymphocytes plays a more dominant role. As a result, it seems reasonable that the PNI, which is a marker consisting of the lymphocyte count and albumin level, may be an indicator of amputation and prognosis. In this study, an association between the lesion location and the PNI was also found. Namely, the PNI value was the lowest and amputation rate was the highest in patients with diffuse involvement.

Although there was no significant difference in terms of comorbidities, the difference in the level of involvement among the patients may have been due to different reasons, such as genetic or individual characteristics.

However, the lower PNI in patients with diffuse involvement may indicate that nutritional disorder and systemic inflammation are impaired more in these patients. Indeed, it is noteworthy that the rate of amputation was higher in these patients. All of these the findings supported the PNI being used as a marker of chronic inflammation and malnutrition, and the diffuse involvement and amputation rates increased in parallel with a low PNI in the PAD patients.

The PNI measurement, which is an inexpensive and easy measurement, especially in patients with prolonged wound healing leading to amputation, can be useful in predicting the prognosis of the patient and intensifying the treatment in clinical practice. While taking measures to prevent amputation, it may be beneficial to identify nutritional supportive approaches to determine the cause of malnutrition in closer follow-up and treatment plans for patients with lower PNI values, and investigate and treat underlying silent chronic inflammation.

This was the first study to demonstrate the utility of the PNI in clinical practice in peripheral artery patients. It is not clear whether the low PNI in patients with limb amputation is the cause or an accompanying finding because it indicates chronic immunonutritional disorder.

The study had some limitations. Although this study was conducted retrospectively, the predictive feature of limb amputation of the PNI in lower-extremity peripheral artery patients suggested an indirect prediction of mortality. Prospective randomized clinical trials will be guiding in terms of possible important features of the PNI in patients with lower-extremity peripheral arteries, as well as all clinical outcomes and prediction of survival and amputation.

## Conclusion

The results indicated that predicting amputation, a condition that affects prognosis in patients with lower-limb PAD, with a simple index to measure, such as the PNI, may be very meaningful in clinical practice. Predicting amputation is of great importance in patients with PAD in terms of prevention, following the patient more frequently and intensifying the treatment. Future prospective studies are needed to test the relationship between amputation predictability as well as other clinical outcomes and mortality.

## Acknowledgments

We would like to thank the participants in the study.

## Competing interest

None.

## Ethical approval

This study was conducted with a prior approval from the Dışkapı Yıldırım Beyazıt Training and Research Hospital Ethics Committee (IEC number: 61/15/ dt.25.03.2019)

## Funding

None.
